# Computing on actin bundles network

**DOI:** 10.1038/s41598-019-51354-y

**Published:** 2019-11-04

**Authors:** Andrew Adamatzky, Florian Huber, Jörg Schnauß

**Affiliations:** 1 Unconventional Computing Laboratory, Department of Computer Science, University of the West of England, Bristol, UK; 2 Netherlands eScience Center, Science Park 140, 1098 XG Amsterdam, The Netherlands; 3 Soft Matter Physics Division, Peter Debye Institute for Soft Matter Physics, Faculty of Physics and Earth Science, Leipzig University, Leipzig, Germany; 4 Fraunhofer Institute for Cell Therapy and Immunology (IZI), DNA Nanodevices Group, Leipzig, Germany

**Keywords:** Computer science, Electronic devices, Computational models

## Abstract

Actin filaments are conductive to ionic currents, mechanical and voltage solitons. These travelling localisations can be utilised to generate computing circuits from actin networks. The propagation of localisations on a single actin filament is experimentally unfeasible to control. Therefore, we consider excitation waves propagating on bundles of actin filaments. In computational experiments with a two-dimensional slice of an actin bundle network we show that by using an arbitrary arrangement of electrodes, it is possible to implement two-inputs-one-output circuits.

## Introduction

An idea to implement a computation by using collisions of signals travelling along one-dimensional non-linear geometries can be traced back to the mid 1960s when Atrubin developed a chain of finite-state machines executing multiplication^1^, Fisher designed prime numbers generators in cellular automata^2^ and Waksman proposed the eight-state solution for a firing squad synchronisation problem^3^. In 1986, Park, Steiglitz, and Thurston^4^ designed a parity filter in cellular automata with soliton-like dynamics of localisations. Their design led to a construction of a one-dimensional particle machine, which performs the computation by colliding particles in one-dimensional cellular automata, i.e. the computing is embedded in a bulk media^5^. Exploring ways to translate the purely theoretical ideas of collision-based computing^6,7^ into nano-computing at a subcellular level, we consider actin networks as ideal candidates for a computing substrate. The idea of subcellular computing on cytoskeleton networks has been firstly announced by Hameroff and Rasmussen in a context of microtubule automata in 1980s^8–10^. Priel, Tuszynski and Cantiello analysed how information processing could be realised in actin-tubulin networks of neuron dendrites^11^. In the present paper we focus purely on actin.

Actin is a crucial protein, which is highly conserved throughout all eukaryotic cells. It is present in forms of monomeric, globular actin (G-actin) and filamentous actin (F-actin)^12–14^. Under the appropriate conditions, G-actin polymerises into F-actin forming a double helical structure^15^. Signals in the actin networks could be represented by travelling localisations. The existence of the travelling localisations — defects, ionic waves, solitons — in cytoskeleton polymer networks is supported by (bio)-physical models^16–25^.

Why is actin more advantageous than other polymers for developing unconventional computers? We provided detailed answers in^26^, which we briefly summarise below. DNA has proven to act well as a nanowire, however no transformations of signals were observed. Tubulin microtubules can act as wires and signal amplifiers, however, there is no experimental evidence of voltage solitons propagating along the microtubules. Actin filaments display very high (comparing to DNA and microtubules) density charges (c. 1.65 × 10^5*e*^/*μ*m) manifested by the extensive charges in electric dipole momentum^27^. Actin filaments also display nonlinear inhomogeneous transmission lines supporting propagation of nonlinear dispersive waves and solitons^17–19,21–25^. Actin can even renew itself via polymerisation and depolymerisation, which can be further tuned with accessory proteins or bionic complexes^28^. On the relevant length scales it is less structurally complex than DNA and therefore experimental prototyping is easier to achieve. Actin is a macro-molecular actuator^29,30^, which opens additional application domains of actin computing circuits — embedded controllers for molecular machinery. Furthermore, the investigated structures are especially suitable since they form by self-assembly settling into an energetic minimum. In this form, the structures can be stable over days even without additional treatment and re-anneal quickly even after harsh mechanical deformations.

Computational studies discussed the feasibility of implementing Boolean gates on a single actin filament^31^ and on an intersection of several actin filaments^32^ via collisions between solitons. Further studies applied a reservoir-computing-like approach to discover functions on a single actin unit^33^ as well as filament^34^. In 2016, for instance, we demonstrated that it is possible to implement logical circuits by linking the protein chains^32^. In such a setup, Boolean values are represented by localisations travelling along the filaments and the computation is realised via collisions between localisations at the junctions between the chains. We have shown that and, or and not gates can be implemented in such setups. These gates can be cascaded into hierarchical circuits, as we have shown on an example of nor^32^.

The theoretical models developed so far address processing of information on a single actin unit or a chain of a few units. Whilst being attractive from a computing point of view, it appears difficult to implement under experimental laboratory conditions. In the present work, we therefore developed an alternative version of the computing on actin networks by considering excitation waves propagating on bundles of actin filaments. Not a single actin filament is considered but an overall ‘density’ of the conductive material formed by the actin bundles arranged by crowding effects without the need for additional accessory proteins^35,36^. First results of this approach are presented below.

## Model

FitzHugh-Nagumo (FHN) equations^37–39^ is a qualitative approximation of the Hodgkin-Huxley model^40^ of electrical activity of living cells:

1 $$\frac{\partial v}{\partial t}={c}_{1}u(u-a)(1-u)-{c}_{2}uv+I+{D}_{u}{\nabla }^{2}$$

2 $$\frac{\partial v}{\partial t}=b(u-v),$$

where *u* is a value of a trans-membrane potential, *v* a variable accountable for a total slow ionic current, or a recovery variable responsible for a slow negative feedback, *I* is a value of an external stimulation current. The current through intra-cellular spaces is approximated by ${D}_{u}{\nabla }^{2}$, where *D*_*u*_ is a conductance. Detailed explanations of the ‘mechanics’ of the model are provided in^41^, here we shortly repeat some insights. The term ${D}_{u}{\nabla }^{2}u$ governs a passive spread of the current. The terms ${c}_{2}u(u-a)(1-u)$ and $b(u-v)$ describe the ionic currents. The term $u(u-a)(1-u)$ has two stable fixed points *u* = 0 and *u* = 1 and one unstable point *u* = *a*, where *a* is a threshold of an excitation.

We integrated the system using the Euler method with the five-node Laplace operator, a time step $\Delta t=0.015$ and a grid point spacing $\Delta x=2$, while other parameters were ${D}_{u}=1$, $a=0.13$, $b=0.013$, ${c}_{1}=0.26$. We controlled excitability of the medium by varying *c*_2_ from 0.09 (fully excitable) to 0.013 (non excitable). Boundaries are considered to be impermeable: $\partial u/\partial {\bf{n}}=0$, where **n** is a vector normal to the boundary.

We used still images of the actin network, produced in laboratory experiments on formation of regularly spaced bundle networks from homogeneous filament solutions^42^ as a conductive template. We have chosen this particular network because it is a result of an experimental protocol which reliably produces regularly spaced aster-based networks formed due to cross-linking and bundling mechanisms in the absence of molecular motor-driven processes or other accessory proteins^42^. These structures effectively form very stable and long-living three-dimensional networks, which can be readily imaged with the confocal laser scanning microscope and subsequently displayed as two-dimensional structures. Thus, these networks can form a hardware of future cytoskeleton based computers^26^.

**Figure 1:**
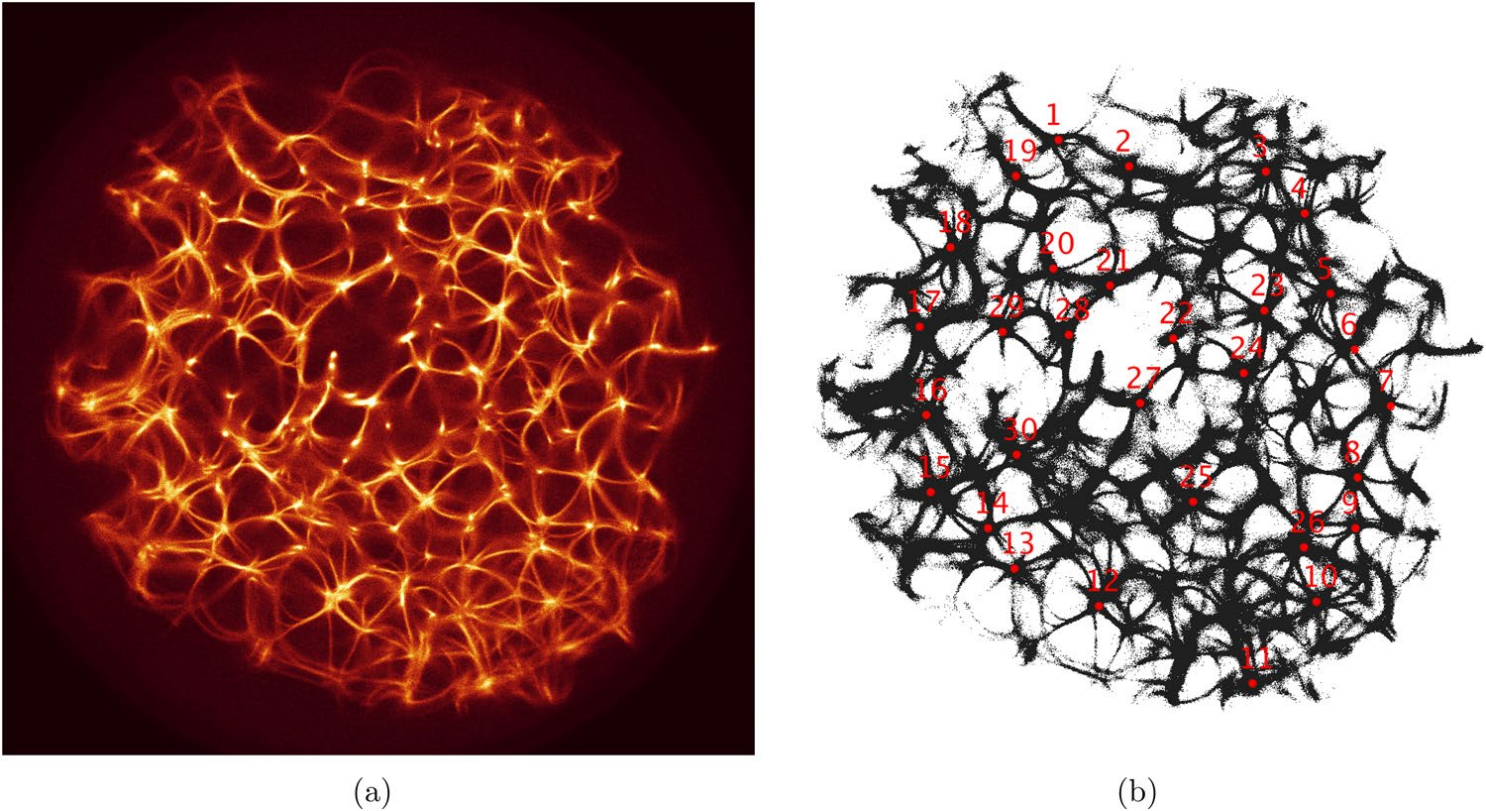
(**a**) Original image, which was published in^42^. (**b**) The ‘conductive’ matrix selected from (**a**) Locations of the electrodes ${E}_{1}\ldots {E}_{30}$ are shown by their indexes.

The actin network (Fig. 1(a)) was projected onto a 1024 × 1024 nodes grid. The original image $M={({m}_{ij})}_{1\le i,j\le n}$, ${m}_{ij}\in \{{r}_{ij},{g}_{ij},{b}_{ij}\}$, where $n=1024$ and $1\le r,g,b\le 255$ (Fig. 1(a)), was converted to a conductive matrix $C={({m}_{ij})}_{1\le i,j\le n}$ (Fig. 1(b)) derived from the image as follows: ${m}_{ij}=1$ if ${r}_{ij} > 40$, $({g}_{ij} > 19)$ and ${b}_{ij} > 19$.

**Figure 2:**
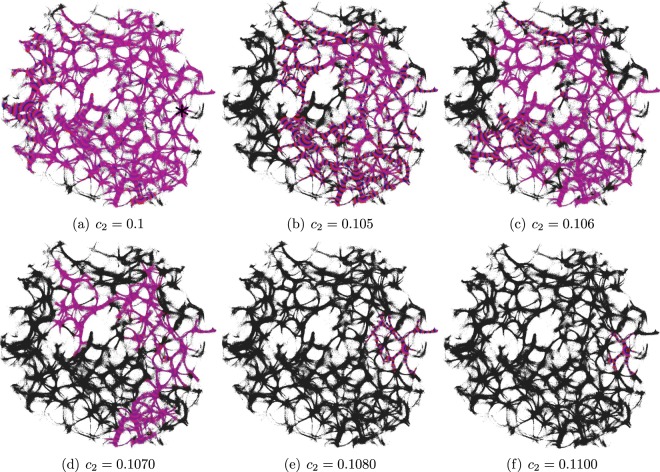
Time lapse images of excitation wave-fronts propagating on the network displayed in Fig. 1(a) for selected values of ${c}_{2}$. In each trial excitation was initiated at the site labelled ‘7’ in Fig. 1(b) and labelled as star in (**a**). Excitation wave-fronts are shown in red, conductive sites in black.

The parameter *c*_2_ determines excitability of the medium and thus determines a range of the network coverage by excitation waves fronts. This is illustrated in Fig. 2.

To show dynamics of both *u* and *v*, we calculated a potential ${p}_{x}^{t}$ at an electrode location *x* as ${p}_{x}={\sum }_{y:|x-y| < 2}({u}_{x}-{v}_{x})$. Locations of the electrodes ${E}_{1},\cdots ,{E}_{30}$ are shown in Fig. 1(b).

The numerical integration code written in Processing was inspired by previous methods of numerical integration of FHN and our own computational studies of the impulse propagation in biological networks^39,41,43–45^. Time-lapse snapshots provided in the paper were recorded at every 150th time step, and we display sites with $u > 0.04$; videos and figures were produced by saving a frame of the simulation every 100th step of the numerical integration and assembling the saved frames into the video with a play rate of 30 fps. Videos are available at https://zenodo.org/record/2561273.

## Results

Input Boolean values are encoded as follows. We earmark two sites of the network as dedicated inputs, *x* and *y*, and represent logical True, or ‘1’, as an excitation. If $x=1$ then the site corresponding to *x* is excited, if $x=0$ the site is not excited. There are several ways to represent output values: presence/absence of an excitation wave-front at a dedicated output site, patterns of spike activity in the network and frequencies of the activity in dedicated output domains. We present four prototypes of logical gates: structural gates (exact timing of collisions between excitation wave-fronts is determined geometrically), frequency-based gates (Boolean values of outputs are encoded into frequencies of excitation), integral activity gates (an activity of the whole network is encoded into Boolean values) and spiking gates (where logical values are represented by spikes or their combinations and a search for the gates is done by using many output electrodes scattered in the network).

### Structural gates

**Figure 3:**
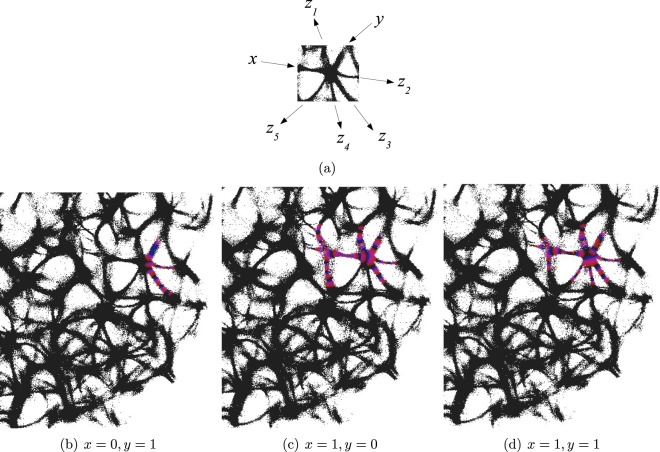
Interaction gate. (**a**) A scheme of input and output channels. (**b**–**d**) Time lapse images of wave-fronts propagating in the gate for inputs (**b**) $x=0$ and $y=1$, (**c**) $x=1$ and $y=0$, (**d**) $x=1$ and $y=1$. Excitability of the medium is ${c}_{2}=0.108$

An example of an interaction gate is shown in Fig. 3. The gate is a junction of seven actin bundles, we call them ‘channels’ (Fig. 3(a)). We earmark two channels as inputs *x* and *y*, and five other channels as outputs ${z}_{1},\ldots ,{z}_{5}$. Such allocation is done for illustrative purposes. In principle, the mapping ${\{0,1\}}^{7}\to {\{0,1\}}^{7}$ can be considered. To represent $x=1$ we excite channel *x*, to represent $y=1$ we excite channel *y*. When only channel *y* is stimulated the excitation wave-fronts propagate into channels ${z}_{2}$ and ${z}_{3}$ (Fig. 3(b)). When only channel *x* is stimulated, the excitation is recorded in channels ${z}_{1}$, ${z}_{2}$, ${z}_{3}$ (Fig. 3(c)). When both channels are excited, $x=1$ and $y=1$, the excitation propagates into all channels (Fig. 3(d)). Thus, the following functions are implemented on the output channels ${z}_{1}=x$, ${z}_{2}={z}_{3}=x+y$, ${z}_{4}={z}_{5}=xy$. The channel ${z}_{1}$ is a selector function. The channels ${z}_{2}$ and ${z}_{3}$ realise disjunction. The channels ${z}_{4}$ and ${z}_{5}$ implement conjunction. An advantage of the interaction gate is that it is cascadable, i.e. many gates can be linked together without decoders or couplers. A disadvantage is that functioning of the gate is determined by exact geometrical structure of the actin bundle network, which might be difficult to control precisely.

### Frequency based gates

**Figure 4:**
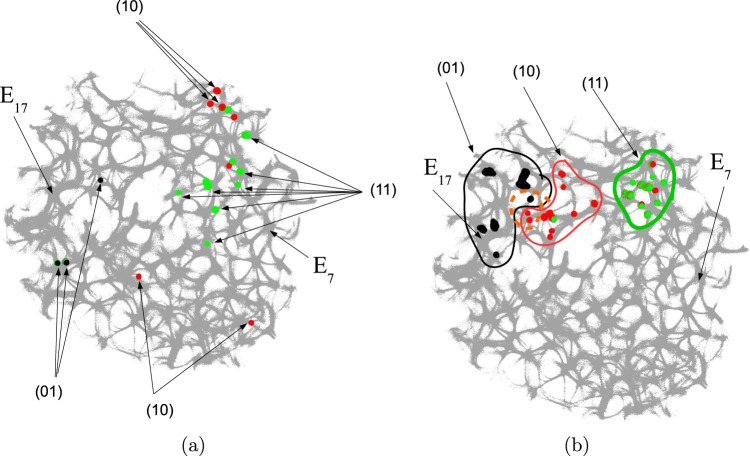
Domains with the highest frequency of excitation represent spatially separated outputs. They are shown by black discs for inputs $x=0$ and $y=1$, red discs for inputs $x=1$ and $y=0$ and green discs for inputs $x=1$ and $y=1$. Inputs *x* and *y* are sites ${E}_{7}$ and ${E}_{17}$ shown by arrows. (**a**) ${c}_{2}=0.1$. (**b**) ${c}_{2}=0.107$.

For each pair of inputs (*xy*) $h\in \{01,10,11\}$ we calculated a frequency matrix ${\Omega }_{h}=({\omega }_{hs})$, $s\in {\bf{L}}$, where each entity with coordinates *s* show how often a node *s* of **L** was excited. At every iteration *t* of the simulation the frequency at every node *s* is updated as follows: ${\omega }_{s}^{t}={\omega }_{s}^{t}+1$ if ${u}_{s}^{t} > 0.1$. When the simulation ends, the frequencies in all nodes were normalised as ${\omega }_{s}={\omega }_{s}/\max \,\{{\omega }_{z}|z\in {\bf{L}}\}$. For each ${\Omega }_{h}$ we selected domains of higher frequency as having entities ${\omega }_{s} > 0.72$. These domains are shown in Fig. 4. This unique mapping allows to implement any two-inputs-one-output logical gate by placing electrodes in the required unique domains. For example, by placing electrodes in the domains which represent outputs for both input pair (01) and input pair (10) (black and red discs in Fig. 4), we can realise a xor gate.

While in excitable mode, ${c}_{2}=0.1$, domains corresponding to different input tuples are somewhat dispersed in the network (Fig. 4(a)), the sub-excitable medium, ${c}_{2}=0.107$, shows compact and well spatially separated domains (Fig. 4(b)). Moreover, for ${c}_{2}=0.107$ we even observe a localised domain (shown by orange dashed contour in Fig. 4(b)), where input tuples (01) and (10) are displayed and thus a xor gate is realised.

### Overall level of activity

**Figure 5:**
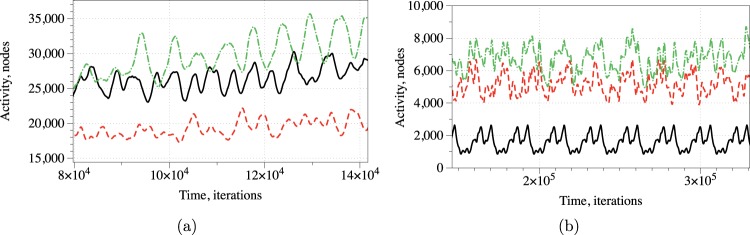
Activity for input pairs (*xy*) (stimulation sites are *E*_7_ and *E*_17_ in (Fig. 1(b))): (01) — black solid, (10) — red dashed, (11) — dashed dotted. (**a**) ${c}_{2}=0.1$. (**b**) ${c}_{2}=0.107$.

At every iteration *t* we measured the activity of the network as a number of conductive nodes *x* with ${u}_{x}^{t} > 0.1$. A stimulation of the resting network evokes travelling wave-fronts, which collide with each other and may annihilate or form new wave-fronts in the result of the collisions. The wave-fronts also travel along cycling pathways in the network. Typically, e.g. after 8 × 10^4^ iterations for ${c}_{2}=0.1$ and 10^5^ iterations for ${c}_{2}=0.107$, the system falls in one of the limit cycle of the overall level of activity with a range of superimposed oscillations (Fig. 5). We found no evidence that shapes of the superimposed spikes in activity reflect the exact combination of inputs, however, an average level of activity definitely does. A correspondence between input tuples and average level of activity *A* as percentage of the total number of conductive nodes is the following:

By selecting an interval of *A*′ as a logical True we can implement a range of gates. Consider the scenario ${c}_{2}=0.1$: *xy* for $A^{\prime} =[0.075,0.085]$, $x\bar{y}$ for $A^{\prime} =[0.045,0.055]$, $\bar{x}y$ for $A^{\prime} =[0.063,0.073]$, $x\oplus y$ $A^{\prime} =[0.045,0.073]$. In the scenario ${c}_{2}=0.107$, a range of gates, implementable by assigning an activity interval to True, is limited to $\bar{x}y$ for $A^{\prime} =[0.005,0.007]$ and $x$ for $A^{\prime} =[0.015,0.025]$.

### Spiking gate

**Figure 6:**
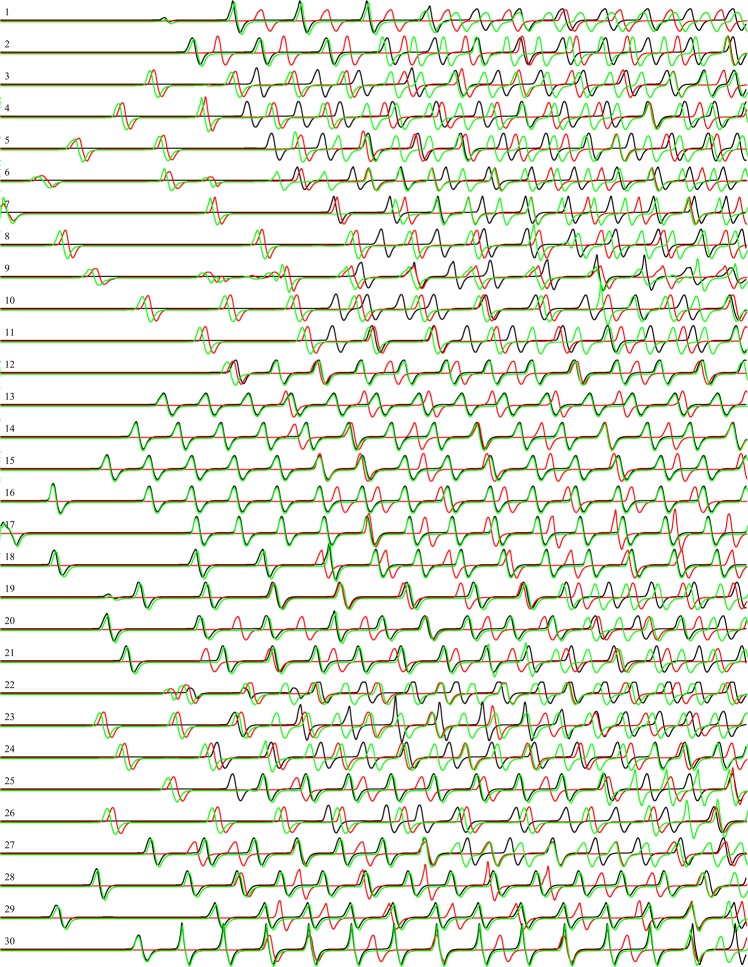
Potential recorded at 30 electrodes (Fig. 1(b)) during c. $14.2\cdot {10}^{4}$ iterations. Indexes of electrodes are shown on the left. Black lines show potential when the network was stimulated by input pair (01), red by (10) and green by (11). Excitability of the medium is ${c}_{2}=0.1$.

A spiking activity of the network shown in Fig. 1(a), with ${c}_{2}=0.1$ in a response to stimulation via electrodes ${E}_{7}$ and ${E}_{17}$ recorded from electrodes ${E}_{1},\cdots ,{E}_{30}$ is shown in Fig. 6. We here assume that each spike represents logical True and that spikes occuring within less than $2\cdot {10}^{2}$ iterations happen simultaneously. Then a representation of gates by spikes and their combinations will be as shown in Table 1.

**Table 1:** Representation of gates by combinations of spikes. Black lines show the potential when the network was stimulated by input pair (01), red by (10) and green by (11).

Spikes	Gate	Notations
	or	$x+y$
	select	$y$
	xor	$x\oplus y$
	select	*x*
	not-and	$\bar{x}y$
	and-not	$x\bar{y}$
	and	*xy*

**Figure 7:**
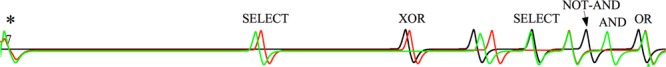
Spikes recorded at the electrode ${E}_{7}$. The moment of initial stimulation is shown by ‘*’. Black lines show the potential when the network was stimulated by input pair (01), red by (10) and green by (11).

By selecting specific intervals of recordings we can realise several gates in a single site of recording. In this particular case we assumed that spikes are separated if their occurrences lie more than 10^3^ iterations apart. An example is shown in Fig. 7.

To estimate the logical richness of the network, we calculated frequencies of logical gates’ discoveries. For each of the recording sites we calculated a number of gates realised during $14.2\cdot {10}^{4}$ iterations (Table 2). In terms of ‘frequency’ of appearance of gates during the simulation, the gates can be arranged in the following hierarchy, from the most frequently found gate to the least frequent gate: select ⊳ {and-not, not-and} ⊳ and ⊳ or ⊳ xor.

**Table 2:** Number of gates discovered for each recording site. Excitability of the medium is ${c}_{2}=0.1$.

*i* (*E*_*i*_)	*x* + *y*	*y*	*x* ⊕ *y*	*x*	$\bar{{\boldsymbol{x}}}y$	$x\bar{{\boldsymbol{y}}}$	*xy*	Total
1	0	7	1	3	2	5	3	21
2	0	8	1	3	2	5	3	22
3	2	2	0	6	5	3	4	22
4	2	3	0	6	5	3	3	22
5	2	2	0	6	4	3	4	21
6	1	4	1	5	4	2	5	22
7	1	4	1	4	2	2	2	16
8	0	3	1	6	3	1	2	16
9	1	2	0	7	3	1	0	14
10	1	2	0	8	4	0	0	15
11	1	3	0	4	3	3	3	17
12	2	7	0	3	0	3	0	15
13	3	10	0	1	0	3	0	17
14	2	11	0	1	0	4	0	18
15	0	11	0	4	0	3	0	18
16	0	11	0	3	1	4	0	19
17	2	9	0	2	0	2	0	15
18	3	8	0	2	0	2	0	15
19	1	5	0	5	2	2	2	17
20	2	8	1	1	1	5	3	21
21	2	7	0	3	1	5	3	21
22	2	4	0	7	2	0	3	18
23	3	5	1	6	2	1	4	22
24	1	5	1	7	2	1	4	21
25	2	7	0	3	2	3	1	18
26	2	2	0	6	5	2	1	18
27	2	6	1	5	2	2	3	21
28	1	10	0	3	0	5	0	19
29	0	9	0	4	0	4	0	17
30	2	9	0	3	0	3	1	18
Average	1.43	6.13	0.30	4.23	1.90	2.73	1.80	18.53
St dev	0.94	3.06	0.47	1.96	1.67	1.46	1.65	2.57
Median	2.00	6.50	0.00	4.00	2.00	3.00	2.00	18.00

The model can realise two-inputs-two-outputs logical gates when we consider values of two recording electrodes at the same specified interval. For example, a one-bit half-adder: one output is and and another output is xor, and a Toffoli gate: one output is select and another xor.

## Discussion

In numerical experiments we demonstrated that logical gates can be implemented in actin bundle networks by various ways of mapping excitation dynamics of the network onto output space. We illustrated the approach with detailed constructions of structural, frequency-based and overall activity based gates. We concluded our study with a comprehensive analysis of spiking gates, where we constructed a frequency of gates hierarchy: select ⊳ {and-not, not-and} ⊳ {or, and} ⊳ xor. The hierarchy matches, with some variations, hierarchies of gates discovered in living slime mould^46^, living plants^47^ and Belousov-Zhabotinsky chemical medium^48^. The gate select is dominating because it reflects a reachability of the recording site from the stimulation site: excitation from one electrode reaches a recording site, while an excitation originated from another electrode does not. The gates and-not and not-and represent the scenario when an excitation wave-front propagating from one input site blocks, for instance by its refractory tail, the wave-front propagating from another input site. A gate and symbolises the situation when wave-fronts originated on both input sites must meet up at some point of their travel to traverse areas with lower excitability, for example loci where a narrow channel suddenly expands. When excitation wave-fronts from both input sites can reach a recording side without annihilating each other, the site implements a gate or. The gate xor reflects the situation when wave-fronts, which originated at different input sites, cancel each other. The presented modelling results are encouraging: they show that a computation can be implemented in an actin bundle network by recording excitation dynamics of the network at few arbitrarily selected domains. The experiments have been conducted with a two-dimensional projection of a slice of a three dimensional actin bundle network. The complete three-dimensional networks will be considered in future studies. Nonetheless, it is important to comment that not dimensionality of the graph but its connectivity might mostly affect a distribution of logical gates. More likely, based on our previous experiments with other disorganised substrates^43,46–48^, the geometry of the gates’ distribution will remain the same.

An experimental implementation of a computing actin bundle network is a challenging task for future studies. Potential realisations of the I/O interface are discussed in our position paper^26^. These include multi-electrode array (MEA) technology^49,50^, as it has been successfully tested with disorganised ensembles of carbon nanotubules^51^, nanofibre light-addressable potentiometric sensor^52^. The inputs can be generated as electrical impulses, conventional for MEA, or using pump-probe approaches^53,54^ or directly exciting polymers into their 350 nm absorption band using Nd:YAG laser^55,56^. Outputs of the actin bundles computer can be recorded via MEA, or by adapting existing system for imaging voltage in neurons^57^, or by single-molecule fluorescence methods such as Förster resonance energy transfer^58–60^.

In living cells, actin networks are highly dynamical systems due to complex interactions with a pool of accessory proteins and continuous energy dissipation through ATP hydrolysis. In contrast to the cellular case, our experimental system was not enriched with any accessory proteins and did not have a constant energy supply. In fact, the arising structures formed solely due to the minimisation of the free energy^42^. Once formed, the networks remained stable over many hours or even days without any additional treatments. We have even observed that harsh mechanical treatments and subsequent bundle breakage are self-repaired by re-annealing effects within the same bundles yielding the very same final network architecture. However, the potential actin dynamics can be even used for our advantage. Dynamical re-configurations could be potentially triggered by releasing, for instance, caged ATP by UV light as an energy source in the system. This can allow implementations of a larger set of logical functions, than a set of functions implementable on a single static network (because a structure of the function is determined by a geometry of the network) and implementation of the evolutionary computing and machine learning techniques. These techniques have been already successfully tested on a thin layer Belousov-Zhabotinsky medium, which is an example of a highly dynamical system expressing continuously changing patters of excitation activity^61–63^. We believe similar techniques could be applied to actin bundle networks in future studies. Moreover, geometry of the networks can be programmed, controlled and sustained as outlined further.

Based on our previous experimental work^42^, we can extend this approach by specifically biasing the architectural design of these networks. Actin in its natural environment has many accessory proteins such as cross-linkers, which directly impact the properties of the bundle structures^64^. We have recently been able to mimic the properties of these naturally occurring accessory proteins with DNA-based, artificial constructs, which can alter the properties of actin structures in a programmable fashion^28^.

The geometrical design of these constructs can be readily tuned by choosing different numbers of binding domains and by altering the underlying DNA template connecting them. These templates can be designed to favour specific binding angles and the number of bundles per junction. They would only act as a molecular precursor for the architecture of the actin system without interfering in the bundle formation and information transport themselves. With the ability to tune the properties of the junctions, we gain control over the computing potential. Theoretically, it would be even possible to mix different types of structural proteins to allow parallel processing of information^65^. Programming a geometry of actin bundles networks with electrical fields^66–70^ could be an approach complimentary to the templating and stabilising the networks with cross-linkers.
